# On The Evolutionary Origin of Symbolic Communication

**DOI:** 10.1038/srep34615

**Published:** 2016-10-10

**Authors:** Paul Grouchy, Gabriele M. T. D’Eleuterio, Morten H. Christiansen, Hod Lipson

**Affiliations:** 1 University of Toronto Institute for Aerospace Studies, Toronto, Ontario, Canada; 2 Cornell University, Ithaca, New York, USA; 3 Columbia University, New York, USA

## Abstract

The emergence of symbolic communication is often cited as a critical step in the evolution of *Homo sapiens*, language, and human-level cognition. It is a widely held assumption that humans are the only species that possess natural symbolic communication schemes, although a variety of other species can be taught to use symbols. The origin of symbolic communication remains a controversial open problem, obfuscated by the lack of a fossil record. Here we demonstrate an unbroken evolutionary pathway from a population of initially noncommunicating robots to the spontaneous emergence of symbolic communication. Robots evolve in a simulated world and are supplied with only a single channel of communication. When their ability to reproduce is motivated by the need to find a mate, robots evolve indexical communication schemes from initially noncommunicating populations in 99% of all experiments. Furthermore, 9% of the populations evolve a symbolic communication scheme allowing pairs of robots to exchange information about two independent spatial dimensions over a one-dimensional channel, thereby increasing their chance of reproduction. These results suggest that the ability for symbolic communication could have emerged spontaneously under natural selection, without requiring cognitive preadaptations or preexisting iconic communication schemes as previously conjectured.

Communication is commonly defined as an event mediated by the use of signs in which the action of an agent or agents (the senders) causes a reaction in one or more agents (the receivers)^1^. Animals communicate using iconic or indexical signals to refer directly to objects or actions^2^, although some species can be taught to use symbols^3^. This type of communication is characterized by a one-to-one mapping between the signal and the object or action. Iconic communication is mediated by signs (icons) which bear a similarity to the form of objects and are immediately recognizable (e.g., as when a monkey opens its mouth and bears its teeth to threaten or when two spots on a butterfly’s wings resemble the eyes of a predator) whereas in indexical communication signs (indices) are only physically or temporally correlated with objects^2^,^4^. For example, to alert other members in their troop of impending danger, vervet monkeys have developed alarm calls directly associated with specific predators or, perhaps more likely, to specific fleeing responses^5^. The vervet monkey alarm calls qualify as indexical, as they do not have any physical similarity with the predators to which they refer. If, however, the alarm for an eagle sounded like (i.e., physically resembled) the call of an eagle, it would qualify as iconic.

By contrast, humans appear to be the only species to have developed a complex system of signs that interrelate with one another. This form of communication—symbolic communication—is based on sign-sign relationships rather than the sign-object relationships of iconic or indexical communication. Symbolic communication relies on one-to-many mappings between a sign or symbol and that to which it may refer. The meaning of symbols depends on a mutually agreed upon cultural context. For example, if one writes “bald eagle,” we know that we are talking about a species of bird of prey. If, however, one writes “eagle eyes,” we are instead talking about good vision. Thus, meaning arises from the sign-sign relationships between “eagle” and the other signs (“bald,” “eyes”) and not from any one sign in particular.

The transition from indexical to symbolic communication is therefore a key evolutionary change because it may signal the origin of language^6^,^7^,^8^ and symbolic thought^2^,^8^,^9^. This transition has not been observed in nature nor has it been reproduced in an artificial evolutionary environment, thus the origin of symbolic communication remains an open problem^10^. Previous works^11^,^12^,^13^,^14^,^15^,^16^,^17^,^18^,^19^ have explored the evolution of communication between robots using evolutionary algorithms. Such approaches employ discrete generations, where offspring replace the entire parent population thus removing the requirement for continuity of an evolving communication scheme between generations. Furthermore, these approaches employ experimenter-defined objective functions (used to evaluate the reproductive viability of individuals or groups of agents) which can restrict the open-endedness of the evolutionary process^20^ and can introduce significant levels of experimenter bias^21^. These experiments have produced a variety of complex communication schemes; however, none has demonstrated the emergence of symbolic communication.

Digital simulation experiments have also been used to explore the emergence of communication. In a simulated world similar to the one presented here, agents evolved indexical communication using a 3-bit channel. Sighted but immobile females used these schemes to direct nearby blind but mobile males to their location on a two-dimensional grid^22^. Avida^23^, a software platform for research on digital organisms, has also been used to study the emergence of experimenter-defined communication schemes among populations of self-replicating computer programs^24^.

## Simulating the emergence of communication

**Figure 1:**
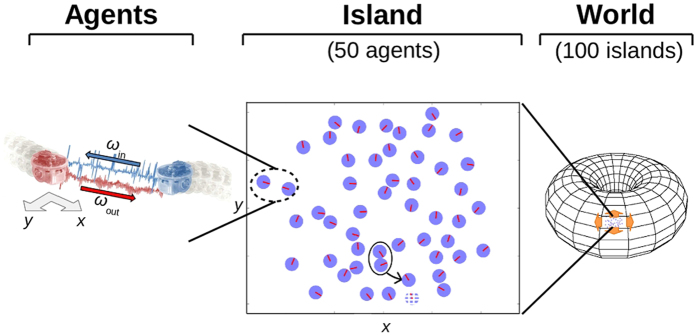
*NoiseWorld*. Robots exist in a 2D world and can sense their own *x* and *y* locations. They cannot sense any information about their neighbours. Robots can produce nondirectional sounds *ω*_out_ and can detect the sounds produced by their nearest neighbour *ω*_in_. Robots live on one of the islands in the world, and when two robots meet, they automatically produce one offspring. A randomly selected robot dies whenever a new offspring robot is born. Islands are organized in a toroid. Offspring robots are occasionally born on one of the four neighbouring islands.

Our evolutionary environment, called *NoiseWorld*, is illustrated in Fig. 1. The agents inhabiting this discrete-time world are represented by evolvable mathematical models^25^ (EMMs) in the form of a system of expandable difference equations which describe the state of the agent, its motor function and its communication output. The use of EMMs enables a direct analysis of a robot’s behaviour by investigating its governing equations.

The structure of EMMs is given by directed tree graphs (see Supplementary Fig. S1), which serve as the agent’s genome, and evolve using the rules of genetic programming^25^,^26^,^27^. Agent genomes are subject to inheritance and mutation. However, no objective fitness function is provided. Two agents sexually reproduce when they are in close proximity (see Methods and Supplementary Fig. S2). Thus *NoiseWorld* evolves asynchronously. Selection pressure arises from the fact that higher reproductive rates lead to lower average agent lifespans as each birth is accompanied by the random death of a robot. This decrease in average lifespan forces less reproductively viable genomes out of the population. There are no objective functions, no discrete generations, and no enforced group selection.

Each robot is supplied with a one-dimensional communication channel over which it emits a signal omnidirectionally and receives a signal from its nearest neighbour. Relative robot positions are recalculated after every timestep, ensuring that a received signal is always arriving from a robot’s current neighbour. Robots cannot detect changes in the identity of their nearest neighbour. Additionally, a third robot has the potential to overhear part of the communications between two other robots: In the situation where two robots share a nearest neighbour, both robots will receive signals from their common neighbour, however the common neighbour will only receive the signal from the robot that is closest to it. Therefore, the closest robot and the common nearest neighbour can signal to one another, while the third robot can only “overhear” one half of the conversation between the other two robots.

Robots cannot determine the direction from which a signal is received nor is there any variation in the intensity of a signal that might otherwise reveal the relative distance of the sender. Any information extracted by the receiver must reside in the content of the signal and not from information inherent in the medium. While the robots are equipped with several mechanical preadaptations–in addition to the availability of a communication channel, the robots know their location and have motor function–no cognitive preadaptation is provided *a priori*.

Since robots share the common goal of reproducing, there is no conflict of interest present, and thus no selection pressure for deceptive communication. Communication schemes that have evolved without a pressure for deception have been observed in nature. For example, there is no conflict of interest during the mating displays that blue-headed wrasses employ to coordinate the simultaneous release of gametes^28^.

## Results

**Figure 2:**
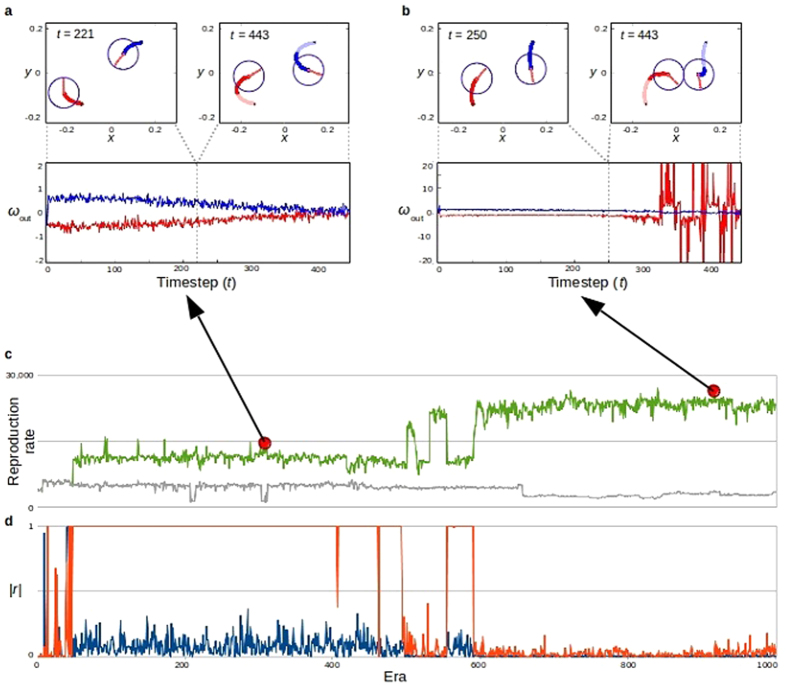
A sample history of an island is examined and top reproducing robots from two different eras are shown interacting. The top frame of these behaviour samples shows the trajectory that the two robots take, while the bottom frame shows their communication outputs *ω*_out_ over time. Auditory interpretations of *ω*_out_ values are provided in Supplementary Audio S1–S2. An era is 100,000 timesteps. (**a**) By era 313, indexical communication has emerged. One can determine directly a robot’s absolute *y* position at a given timestep from its *ω*_out_ value (*y* = *ω*_out_/4.36, see text). (**b**) By era 937, symbolic communication has emerged. Robot position information can no longer be determined from observing single *ω*_out_ values. Instead, relative robot positions are revealed through sign-sign relationships (i.e., by observing both agents’ *ω*_out_ values, see text). (**c**) Reproduction rates are shown with (green) and without (grey) communication enabled. (**d**) Also shown are the magnitudes of the Pearson product-moment correlation coefficients between the position (*y* in red, *x* in blue) and *ω*_out_ of each era’s most reproductively successful agent.

Figure 2 shows a sample history of one simulation run (see Supplementary Fig. S3 for others). Population “snapshots” were taken once per era (defined as 100,000 timesteps). As the simulation begins, there is no communication on the island because an enabled communication channel does not provide any reproductive benefits (see Fig. 2c). Moreover, at era 48 for example, *ω*_in_, the variable containing a neighbour’s output signal, appears nowhere in an agent’s governing equations (see Supplementary Equations S1–S3). Robots cannot respond to incoming signals and thus reproduction occurs only by chance. As time progresses, the population’s reproductive success improves with the first stepwise increase occurring at about era 50. At this point, initial communication emerges; statistically, as measured by the Pearson correlation coefficient, the robot communication output signals (*ω*_out_) are found to be highly correlated with their latitude (*y*) as shown in Fig. 2d. Indeed, for an example agent at era 313 (see Fig. 2a), the output signal equation (see Supplementary Equations S4 and S5) is


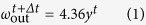


exhibiting a direct one-to-one relationship. This is indicative of an indexical communication scheme. Just as different vervet monkey alarm calls indicate the presence of specific predators, robot *ω*_out_ values indicate specific robot *y* positions. By era 600, reproduction rates have jumped to a new plateau, whereas the correlation between output signal and robot position has dropped precipitously. Agent genomes reveal that the output signals involve both latitude and longitude but there is no longer a one-to-one relationship between signal and robot location. For example, at era 937 (see Fig. 2b) a typical output signal equation (see Supplementary Equations S6–S7) is


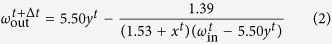


A given value of *ω*_out_ corresponds to multiple (*x*, *y*) locations. It is a one-to-many relationship. Equally important, an input communication signal *ω*_in_ appears in the equation: the output is modified by the input, indicating a dialogue. This dialogue resolves the ambiguity in the signal’s meaning, implying a sign-sign relationship in contrast to the sign-object relationship evident earlier in the evolutionary process. A qualitative transition in the communication scheme has clearly occurred. The indexical mode of communication has evolved into a rudimentary form of symbolic communication.

A typical equation determining the orientation of the robots employing indexical symbols is


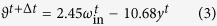


The equations determining the orientation of the symbolically communicating robots are structurally similar:


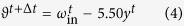


This structural similarity between these two species suggests that the complexification of the signal outputs towards symbolic communication was able to yield reproductive benefits by exploiting the minimal preexisting cognitive machinery necessary for indexical communication. The probability of these changes occurring during a single reproductive event is small considering the simulation’s mutation rates (see Methods). It is far more likely that this transition occurred via a series of mutations and/or sexual recombination events that were able to produce reproductively viable transient communication schemes that exploited preexisting listening capabilities. Future step-by-step documentation of this transition will require snapshots taken at a frequency several orders of magnitude higher than was used to capture the data presented here.

**Figure 3:**
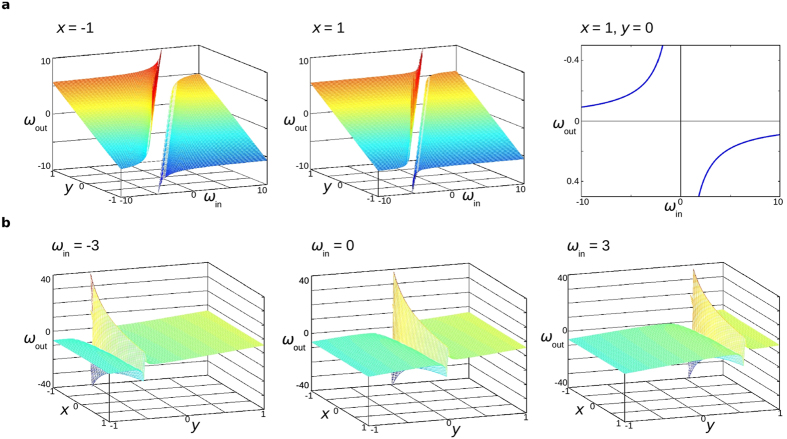
Various visualizations of the evolved communication output *ω*_out_. (**a**) Neighbouring robots determine relative *y* positions via their *ω*_out/in_ (i.e., dialogue) values oscillating between the two separate regions shown here. The *ω*_out/in_ of the robot with the higher *y* value will settle in the left region (resulting in a higher *ω*_out_), while the other settles in the right region (resulting in a lower *ω*_out_), thus “deciding” relative north/south robot position. (**b**) As the two robots approach a common *y* position (the nonlinear part of these plots), the robot with the smaller *x* position will see the magnitude of its *ω*_out_ increase faster than that of its neighbour, which in turn forces the neighbour’s *ω*_out_ back towards linear behaviour, thus “deciding” their relative east/west position.

From a communications perspective, the dialogue between two robots from era 937 reveals how they are able to negotiate a meeting. They first resolve their difference in latitude (*y*). When a robot’s (the sender’s) output signal is larger than its neighbour’s, *ω*_out_ > *ω*_in_ from the sender’s perspective, it is communicating that the sender is north of the receiver (see Figs 2b and 3a). The resulting action is for the sender to move south and the receiver to move north. As the two robots converge upon a common latitude, the magnitude of their signals begins to increase. Smaller values of *x* translate to faster increases and larger communication output signals force the receiver back towards smaller outputs, thus “calculating” relative robot *x* positions (Fig. 3b). After this “discussion,” larger output signals indicate that the sender is “west” of the receiver.

The meanings inferred by one robot in the other’s signals emerge from sign-sign relationships. For example, if we only observe small communication output signals from a single robot, we would not be able to discern if this agent were indicating north, south or east (this is analogous to the “eagle” example given above, where one word/sign is not enough to resolve meaning). Since third parties may overhear one half of a dialogue between two robots, there is a pressure to prevent eavesdroppers from extracting the location of a potential mate from one side of the conversation, as otherwise they might reach this mate first. This pressure may play a role in the emergence of these symbolic communication schemes where meaning cannot be extracted from a single robot’s signals, although further experimentation is required to confirm this.

**Figure 4:**
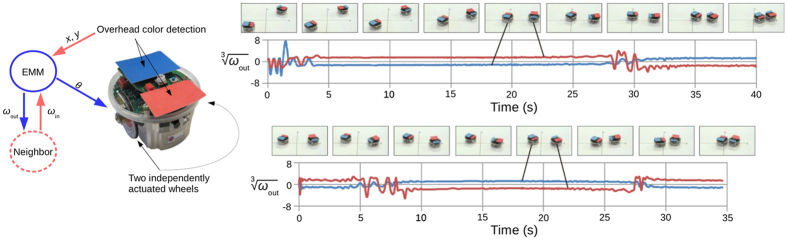
The genome of an agent with an evolved symbolic communication scheme is embodied in two e-puck robots. Agents are supplied with their position information via an overhead webcam and colour detection software. Evolved agents are run on a laptop (not shown) that handles communication between agents and sends instructions to the robots via Bluetooth. Two hardware experiments are shown, with images taken at 5 second intervals shown in the first row, and the corresponding inter-robot communication data shown underneath. Auditory interpretations of *ω*_out_ values are provided in Supplementary Audio S3–S4.

These sign-sign relationships are abstract; that is, what they describe cannot be sensed directly by the robots (in this case, they cannot directly sense relative position). Furthermore, these relationships are arbitrary; that is, alternative meanings can and have emerged from other evolutionary runs. The example above produces a primarily “north/south” movement and is reflected in the communication patterns which evolved; other populations, however, have evolved to use an inverted “south/north” communication scheme or even a primarily “east/west” system (see Fig. 4, Supplementary Figs S4–S8 and Supplementary Equations S8–S54).

## Discussion

These results provide a new window on a potential pathway for the emergence and evolution of symbolic communication (see Supplementary Video S2), one that does not require preexisting brains with a high degree of complexity as previously conjectured^29^. They moreover demonstrate an unbroken evolutionary pathway to simple symbolic communication via indexical communication without the need for iconic communication, which has previously been proposed as a possible evolutionary stepping stone to symbolic communication^30^. Simple indexical communication strategies similar to the one described above emerged in 99 of 100 simulation runs where the communication channel was enabled (10 additional runs were performed with the communication channel disabled). Nine of these populations evolved further, developing a rudimentary form of symbolic communication, as indicated by the sign-sign relationships on which these communication schemes rely^2^,^4^ (see Table 1 and Supplementary Table S1). Reality is of course much more complex than these simulations, as organisms in nature do not typically know their absolute coordinates and natural communication systems contain inherent physical information. Moreover, human symbolic communication is largely learned, whereas here behaviours, including communication schemes, are genetically encoded. Therefore, while these results demonstrate one possible pathway to symbolic communication via indexical communication and without substantial preexisting cognitive complexity, this is not necessarily the evolutionary path that human communication took, nor does this prove that symbolic communication cannot emerge from iconic communication or cognitive preadaptations. Nonetheless, these results demonstrate that simple symbolic communication can emerge spontaneously from a population of initially noncommunicating embodied agents in a relatively short evolutionary timespan given a limited capacity communication channel, no conflict of interest, and a selection pressure for cooperation. The role of overheard signals in shaping these symbolic communication schemes and the potential effects of deceptive signals on the evolutionary process are left to future work.

Given the cognitive simplicity of these symbolically communicating robots, one cannot help but wonder why humans are the only species to have evolved symbolic communication in nature. One possible explanation is that the combined dimensionality of both verbal and nonverbal animal communication media, as well as their inherent information (such as directionality), provide sufficient information transmission capacity for animal species’ communication needs. We might also speculate that perhaps similar symbolic systems do exist in other species but that we have not yet discovered them. However, it may require the combination of other specifically human skills that enable cultural evolution for such a simple system to be elevated into the kind of complex communication system we observe in human language.

## Methods

### Evolvable mathematical models

The evolvable mathematical models (EMMs) used to represent the agents are defined by a system of equations of the form


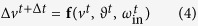



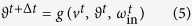



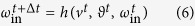


where *v*^*t*^ is the state vector of the agent at time *t*, *v*^*t *+ Δ*t*^ = *v*^*t*^ + Δ*v*^*t+*Δ*t*^ at the next timestep, *ϑ*^*t*^ is the motor output governing the direction of the robot’s movement relative to a given reference direction and *ω*^*t*^_in/out_ are the robot’s input (from the nearest neighbour) and output communication signals. Every state vector includes the coordinates *x*^*t*^, *y*^*t*^ of the robot. These equations are encoded in a set of directed tree graphs which serves as the agent’s genome (see Supplementary Fig. S1). Terminal nodes of the equation trees take on the values of one of the variables (variable leaves) or a numerical constant (constant leaves) while nonterminal (branch) nodes perform one of the four basic arithmetic operations (addition, subtraction, multiplication, division). We use the term “evolvable mathematical model” to refer to the genomic representation of agents by equation trees as evolved via genetic programming.

### *NoiseWorld*

When two robots come into close proximity to each other (within a prespecified “reproduction distance” *ρ*, here *ρ* = 0.139), an offspring is born by sexual reproduction using genetic programming. During reproduction, offspring genomes are subject to a variety of genetic operators. For each equation that the two parents have in common (the equations have unique identification tags based on when they first appeared via mutation in the simulation), either the equation from parent 1 or parent 2 will go to the offspring. Which equation is inherited is decided randomly for each equation in common. An offspring must receive at least one equation from each parent, thus sexual reproduction is enforced at the equation level. If an offspring receives an equation that contains a variable modified by another equation that is not common to both parents, the offspring will inherit that equation as well (see Supplementary Fig. S2 for several examples).

A mutation will occur in an equation tree with a probability of *p*_*m*_; here *p*_*m*_ = 0.025*β*/*n*, where *n* is the number of trees in the genome and *β* is independently calculated on each island every 10,000 timesteps as 500/*b* with *b* being the numbers of births on the island in the previous 10,000 timesteps. The parameter *β* saturates at 100 but has no minimum; it is used in an effort to keep the number of mutations per unit time constant.

A tree mutation is a point or subtree mutation with equal probability. A point mutation takes the form of a perturbation of a constant leaf (if any exist in the tree) or the mutation of another node with equal probability. A perturbation of a constant is drawn from the Guassian distribution N(*μ*, *σ*); here, *μ* = 0 and *σ* = 0.5. A mutation to a branch-node reassigns it to another arithmetic operation and a mutation to a variable leaf changes it to another variable or a new constant, *k*; here, *k* ∈[−5, 5]. A subtree mutation replaces a randomly selected node with a randomly generated subtree (generated via the ramped half-and-half method, see below). There is a 5% chance that the randomly generated subtree will replace the entire original tree, with the original tree then being spliced onto a randomly selected node on this new subtree. A genetic splice operation occurs with a probability *p*_*m*_ whereby a randomly selected node is replaced with a randomly selected subtree from a parent genome.

Initial conditions of the state, *v*^0^, are also subject to mutation with probability *p*_*m*_; in these mutations, the initial state values are either augmented by a perturbation taken from N(0, 0.25) or completely replaced with a random value drawn from the interval [−1, 1] with equal probability.

Finally, for each tree in an offspring genome, there is a probability 0.5*p*_*m*_ that a new state equation will be added to the offspring’s genome, with a reference to the corresponding new state variable inserted into a randomly selected location on the tree. The equation tree for the new variable is initialized in the same manner as for the primordial population (see below).

There is a total of 100 islands in *NoiseWorld*, each with a large two-dimensional expanse (−20 < *x* < 20, −20 < *y* < 20). If a robot reaches the edge of an island (which is only possible with very long lifespans as robots are initialized far from the edges of their island and can only move an average of 0.0005 units per timestep, see below), it effectively falls off the island, a death that is enforced at the next reproduction event. The subpopulations on each island are isolated except for the occasional migration occurring at birth (see below). Topologically, *NoiseWorld* is toroidal where every island is surrounded by eight neighbouring islands, four sharing a “border” and four sharing a “corner” on a two-dimensional manifold. Each island is seeded with 50 agents and each agent is initialized with a random genome using the “ramped half-and-half” method^27^ to generate trees with a maximum depth of 1 or 2. Ramped half-and-half is a combination of two methods, the “full” method and the “grow” method. In the “full” method, nonterminal nodes are randomly generated until the maximum depth is reached. At the maximum depth, only terminal nodes are created. In the “grow” method, as in the “full” method, only terminal nodes are created at the maximum depth. The difference is that before the maximum depth is reached, randomly generated nodes can be either terminal or nonterminal nodes, allowing for a wider range of potential tree shapes. The ramped half-and-half method chooses to create a random subtree using either the “full” or “grow” method with equal probability.

Each agent is supplied with two immutable equations, Δ*x*^*t*+Δ*t*^ = *a*Δ*t* cos*ϑ*^*t*^ and Δ*y*^*t*+Δ*t*^ = *a*Δ*t* sin*ϑ*^*t*^ in Δ*v*^*t*+Δ*t*^, which govern its movement; *a* is drawn from N(1, 0.025) and Δ*t* = 0.0005 in dimensionless time units. Each robot knows its latitude and longitude, *x* and *y*, but has no direct information about any of its fellow robots. The angle *ϑ*^*t*^ is measured relative to either “east” (+*x* direction) or “north” (+*y* direction).

Offspring begin life in a randomly selected location on its parents’ island (within a circle, here of radius 1.13, centred on the origin) although there is a small probability (*p*_*b*_ = 0.001) that it will appear on a bordering island (diagonal migrations are not permitted). A minimum distance (here the reproduction distance *ρ*) to the offspring’s nearest neighbour is enforced. Parents are also moved to new random locations on their island (in the same manner as described above for their offspring) and reinitialized. Migration allows the spread of genes among islands. Otherwise, robots are restricted to remain on their native islands. To maintain a constant population, when a birth occurs, another robot randomly dies.

A newly created offspring genome has a 10% chance of being selected to undergo equation reduction. In such an event, the following operations are applied recursively across all of the agent’s equation trees:

- The subtraction, addition, multiplication or division of two constants is reduced to a single constant by performing the encoded operation.
- The sum of two identical subtrees is reduced to 2× a single version of the subtree.
- The subtraction of two identical subtrees is reduced to 0.
- The multiplication of a subtree by 0 is reduced to 0.
- The division of 0 by a nonzero subtree is reduced to 0.

An agent’s genome is limited to a maximum of 200 nodes across all of its equation trees. An offspring born with more than 200 nodes dies immediately.

If one or more of an agent’s output variables exceed the minimum or maximum representable floating-point number, the agent will have that output set to a random floating-point number and will be selected to die when the next birth occurs.

It is worthwhile noting that further investigation and observation of the robots’ behaviour show the evolutionary process to be developing a simple control mechanism. Taking again the evolved agent of era 937 as our example (see Supplementary Equations S6–S7), we see that the mutual dynamics of two identical agents (1 and 2) possesses the fixed point *x*_1_ = *x*_2_, *y*_1_ = *y*_2_. Moreover, this point behaves in a stable fashion. From a control-theoretic viewpoint, then, the evolution produces a stable controller in which the objective is bring two agents to consensus in position and where *ϑ*^*t*^ is the control variable and *ω*_out/in_ serves as the measurement variable.

### Computational experiments

All simulation experiments were run for 48 wall clock hours on a dedicated Linux server with an Intel Xeon E5540 at 2.53 GHz. Each island is implemented as a separate process so that the algorithm can take full advantage of the parallel architecture of the Intel Xeon CPU (8 cores/16 threads). A master/slave parallel implementation is used, where a “master” process handles the synchronization of “slave” processes (i.e., the islands). Islands are synchronized and migrants exchanged every 10,000 timesteps. Islands introduce incoming migrants into their subpopulations at a rate of *η* =10,000 migrants per timestep (in a randomized order). Migration events are treated as new births on the receiving island, thus engendering a random death on the island at the following timestep. While migration isn’t necessary for symbolic communication to emerge, it has the effect of improving the probability of a run achieving symbolic communication, as well as reducing the accumulation of neutral mutations in agent genomes, thus significantly increasing the number of eras that can be simulated in 48 wall clock hours (Supplementary Table S2).

To test how an island snapshot performs with and without communication enabled (i.e., *ω*_in_ = 0), as well as to collect data for the correlation calculations (Fig. 2d and Supplementary Fig. S3), a control test simulation was performed. The duration of a control test run is one era (100,000 timesteps) and the genomes used are taken from a snapshot of an island population. The robots are initially placed randomly in the test world and initialized. If during the control test run two robots meet one another (within the distance *ρ*, see above), the event is counted as a reproduction event but no offspring genome is created. Instead, the two parent robots are moved to new random locations and reinitialized. This prevents any evolution during the control runs. The effects of births and deaths were simulated by moving a robot to new random position and reinitializing it with a probability of 0.001 per robot per timestep.

For correlation calculations, the communication outputs and position information of the top reproducing agent in the snapshot are recorded throughout the test simulation, yielding 100,000 sets of input/output values per test. The Pearson product-moment correlation coefficient *r* (“correlation” in Fig. 2d and Supplementary Fig. S3) was calculated as


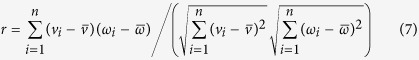


where *n* is the number of samples (*n* = 100,000), *v*_*i*_ is a sample of the input variable in question (i.e., *x* or *y* position), 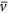 is the mean of the input samples, *ω*_*i*_ is a sample of the communication output, and 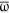 is the mean of the communication output samples. If the communication output is a constant then *r* is undefined and these points are omitted from Fig. 2d and Supplementary Fig. S3.

### Source code

The source code for the computer simulation experiments described in this work is freely available at https://github.com/pgrouchy/NoiseWorld.

### Embodied robotic agents

The hardware experiments in this work were performed on e-puck robots. Two agents (EMMs) were run in a synchronized fashion on a laptop, with motor speed adjustments being sent to two e-puck robots via Bluetooth.

Robot orientation *ϑ* and positions *x* and *y* were determined using an overhead webcam (640 × 480 resolution), colour detection software and coloured markers affixed to the top of the robots (see Fig. 4). Binary images indicating the locations of the red or blue markers were created from webcam images by using RGB colour masks. Blob detection was then performed on these binary images. The pixel values of the centroids of the two largest blobs were used to determine robot position, with the larger of the two red blobs always indicating one robot, and the larger of the two blue blobs indicating the other robot. Pixel values of blob centroids were scaled by 424 to yield position value magnitudes comparable to those that agents would typically see in simulation. A robot’s orientation was determined by taking the arctangent (using the atan2 function) of the difference between the pixel values of the centroids of its two blobs/markers.

The protocol for the robotic experiments was as follows (Supplementary Fig. S9):

1. Set all agent variables to their initial values.
2. Get agent *x* and *y* positions from overhead tracking system.
3. Evaluate both sets of agent equations for 10 timesteps, with an agent’s *ω*_in_ being set to the other agent’s *ω*_out_ from the previous timestep. The *x* and *y* input values are not changed during these 10 timesteps, although new noise values are used at each step; *ϑ* is treated as an internal variable and is thus updated at each step.
4. Calculate the cumulative expected motion of each agent over the past 10 timesteps. This yields a new expected position. Each robot is turned to face its expected position (±π/16) and then set to drive forward. If a robot is already within ±π/16 of this expected orientation, it is not turned. If the robots are in motion and at least one agent needs to turn, both robots are stopped. Otherwise they are left to continue forward in their current direction.
5. Loop back to step 2.

For the hardware experiments presented in this paper, the following EMM was used for both agents:


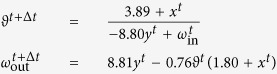


This is the top reproducing agent from a previously unreported run.

### Auditory interpretations of communication outputs

Auditory interpretations of communication outputs (Supplementary Audio S1-S4) were generated using SuperCollider. All communication outputs are converted to a frequency using the following equation: *freq* = 14(*ω*_out_ + 25), with 210 ≤ *freq* ≤ 490. Values that fall outside of this range are set to the nearest boundary value. In the case of Fig. 2a, frequencies were produced as follows: *freq* = 140(*ω*_out_ + 25) − 3150. Each timestep’s sounds (i.e., the frequency interpretations of the communication output of each of the two agents being tested) last for 0.05 s.

## Additional Information

**How to cite this article**: Grouchy, P. *et al*. On The Evolutionary Origin of Symbolic Communication. *Sci. Rep.* **6**, 34615; doi: 10.1038/srep34615 (2016).

## Supplementary Material

Supplementary Information

Supplementary Video 1

Supplementary Video 2

Supplementary Video 3

Supplementary Video 4

Supplementary Video 5

Supplementary Video 6

Supplementary Data 1

Supplementary Data 2

## Figures and Tables

**Table 1:** Summary of results.

Comm. type	Number of runs	Max. isl. repro. rate (per era)			Number of eras		
		μ	σ	Rank-sum	μ	σ	Rank-sum
Symbolic	9	23890.11	1044.48	*P* < 0.00001	1234.00	173.91	*P* = 0.12215
Indexical	90	20030.89	1510.12	*P* < 0.00001	1373.19	296.99	*P* = 0.09423
None	11	7559.55	409.96		1222.36	242.98	

Runs are divided into three types based on the highest level of communication that emerged (“none” in the case of one run where communication never emerged and 10 additional runs with *ω*_in_ = 0 for all *t*). The average (*μ*), standard deviation (*σ*), and two-tailed Wilcoxon rank-sum test *P* values are shown.
