# Supplementary material for: On The Evolutionary Origin of Symbolic Communication
Source: Sci Rep. 2016 Oct 10;6:34615. doi: 10.1038/srep34615 (PMC5056373; doi:10.1038/srep34615)
Supplement: Supplementary Information [file srep34615-s1.pdf]

On the evolutionary origin of symbolic communication

Paul Grouchy[1,2], Gabriele M. T. D'Eleuterio[1], Morten H. Christiansen[2], and Hod Lipson[2,3,*]

Supplementary Information

*Corresponding Author:

Hod Lipson, PhD
Prof. of Mechanical Engineering, Columbia University in NYC
Office: 535E Mudd Building, 500 W. 120th St., New York, NY 10027 USA
Mailing address: 500 W. 120th St., Mudd 220, New York, NY 10027 USA
Cell: +1 (607) 592 4383 Email: hod.lipson@columbia.edu
hodlipson.com

[1]University of Toronto Institute for Aerospace Studies, Toronto, Ontario, Canada
[2]Cornell University, Ithaca, New York, USA
[3]Columbia University, New York, New York, USA

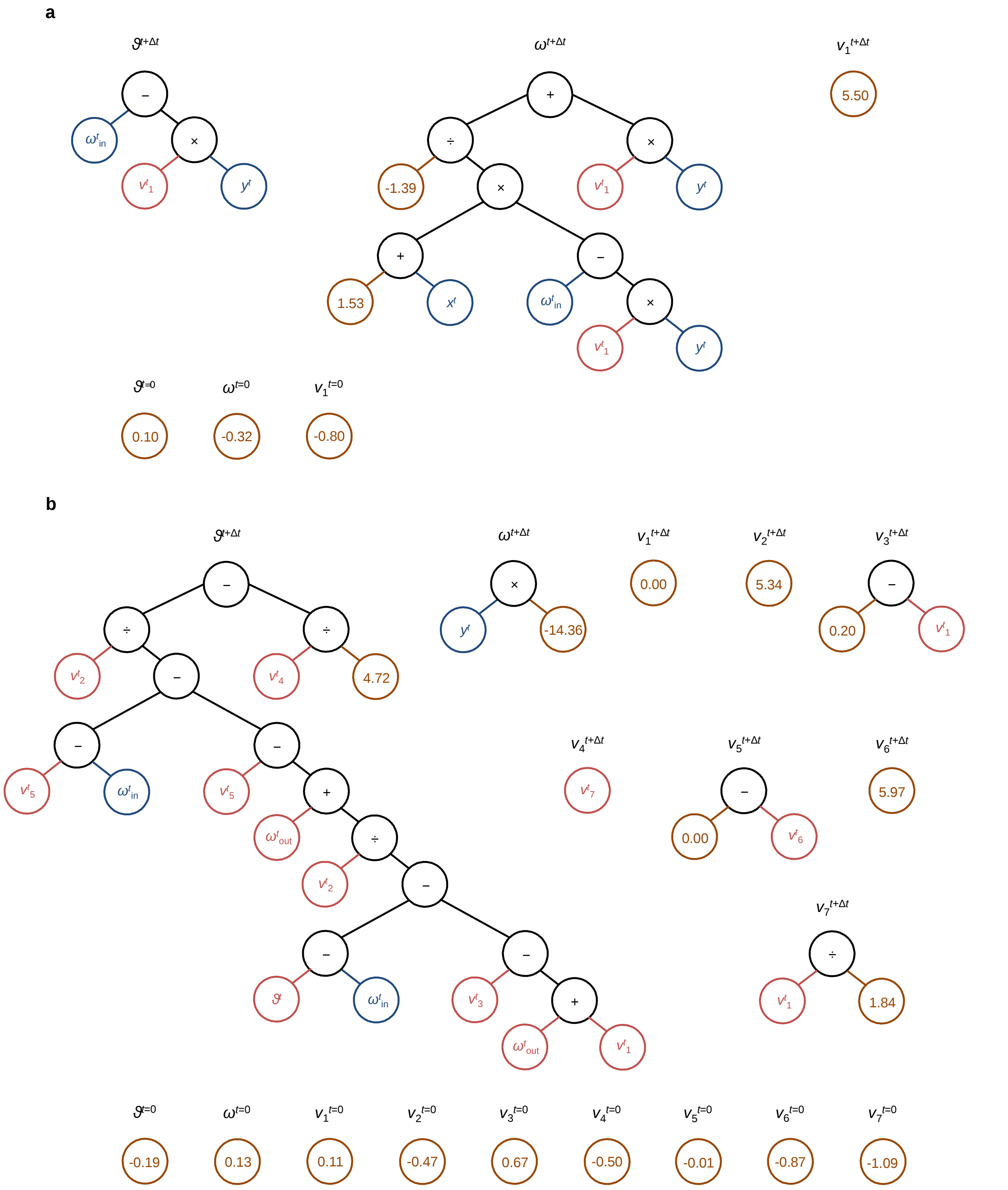

Figure S1. Agent genomes. Two complete EMM genomes are shown, including evolved initial conditions. (**a**) The genome of the top agent from run 7 (see Supplementary Supplementary Table S1 online online). Note that this agent uses a symbolic communication scheme. (**b**) The genome of the top agent from run 81 (see Supplementary Supplementary Table S1 online online). Note that this agent uses an indexical communication scheme.

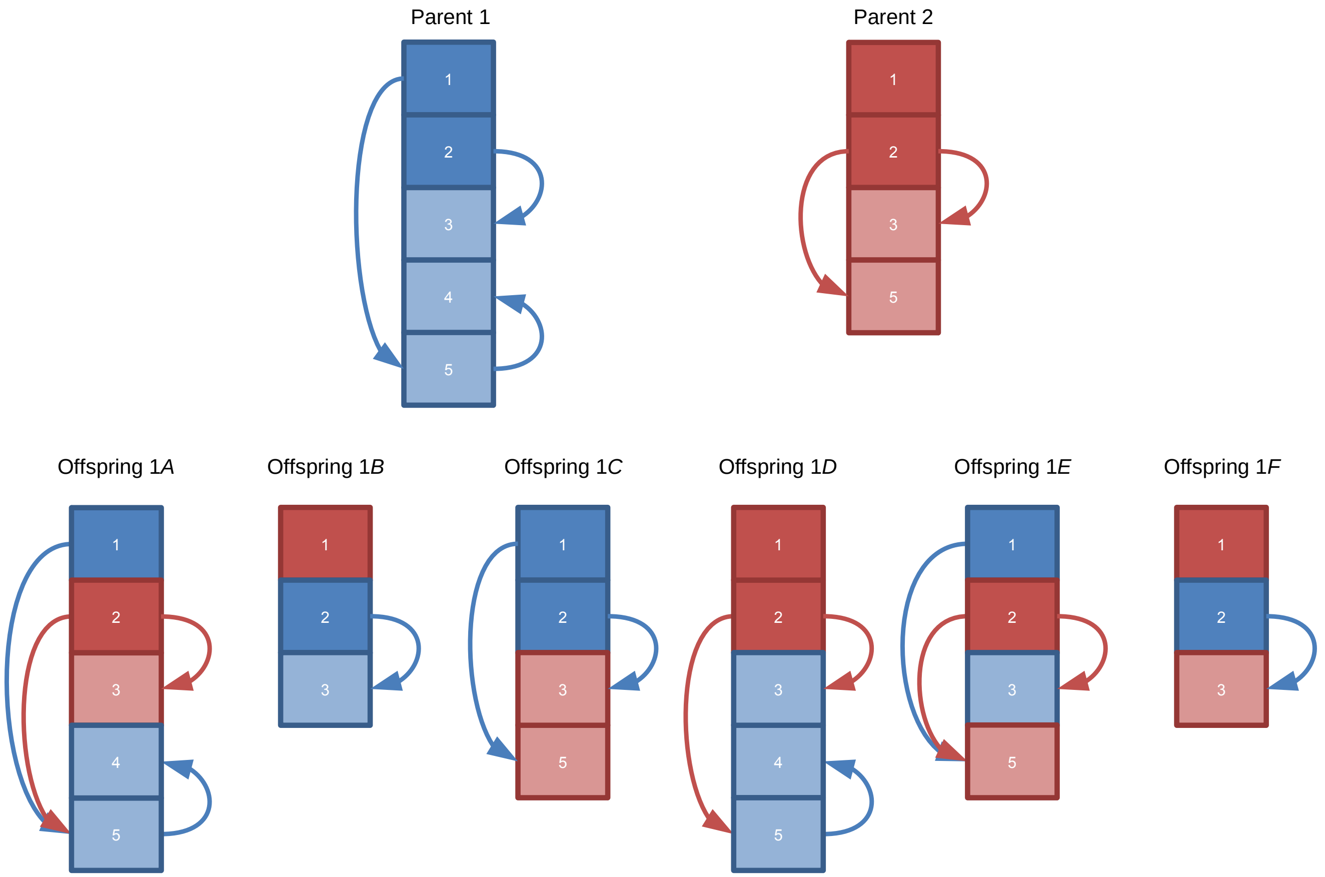

Figure S2. Sexual reproduction. Several potential offspring genomes that can be produced from Parent 1 and Parent 2 reproducing. This diagram assumes that the agents have 2 outputs. Each rectangle represents a genetically-encoded equation and its ID. Lighter coloured rectangles are extra variables/equations that do not affect outputs directly. Arrows show which equations depend on which extra variables/equations. Note that if there are no equations that depend on an extra variable in an offspring genome, that extra variable and its corresponding equation are discarded.

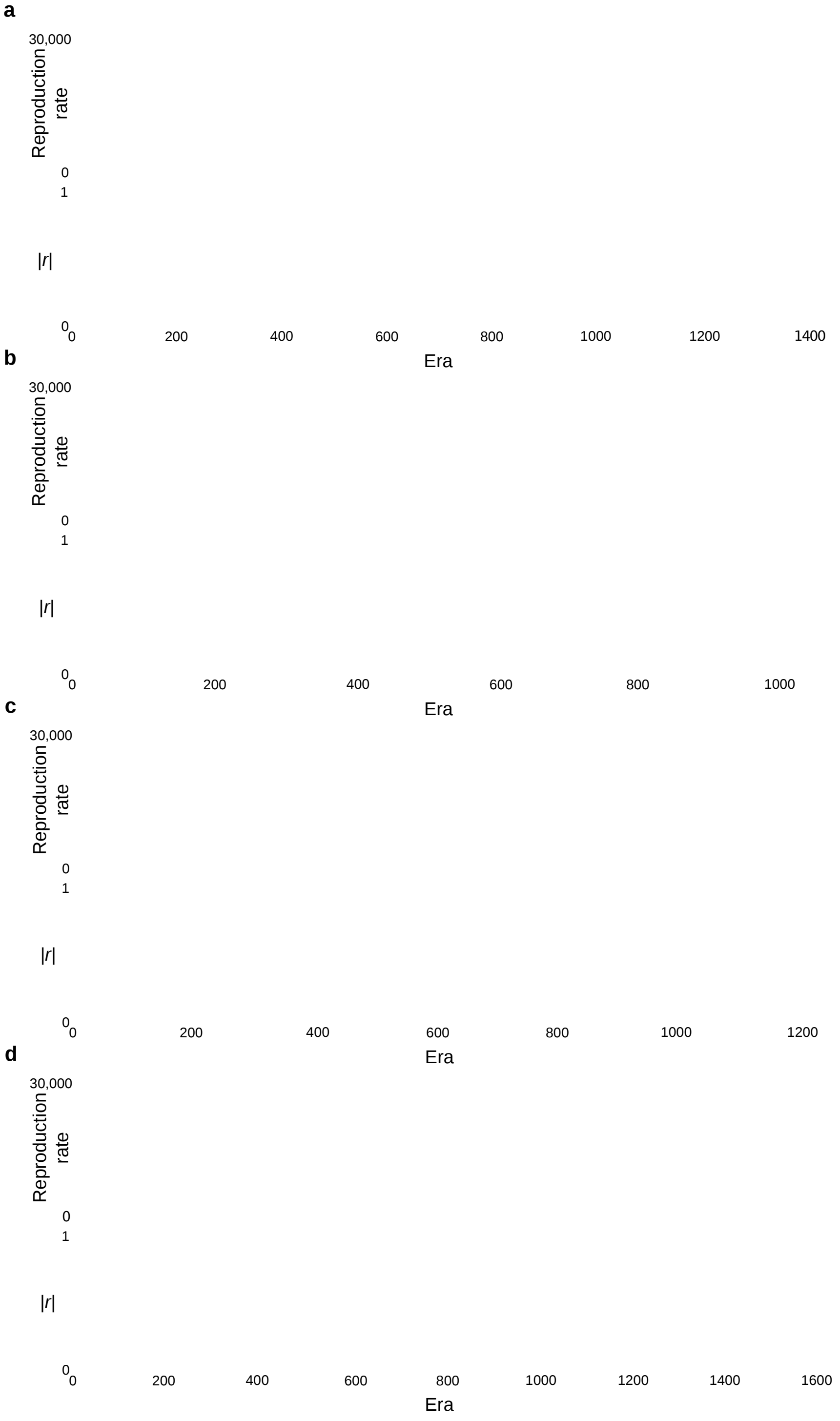

Figure S3. Sample histories of additional evolutionary runs. Reproduction rates are shown with (green) and without (grey) communication enabled. The magnitude of the Pearson product-moment correlation coefficient between the top agent's position (y in red, x in blue) and ω_{out} is also shown (see Methods). (**a**) The full evolutionary history of the top island from run 7 (see Supplementary Supplementary Table S1 online online) is shown. Note that the symbolic species is pushed out by an indexical one (which has a worse reproduction rate) late in the simulation. (**b**) The evolutionary history of the top island from run 24 (see Supplementary Supplementary Table S1 online online) is shown. (**c**) The evolutionary history of the top island from run 70 (see Supplementary Supplementary Table S1 online online) is shown. (**d**) The evolutionary history of the top island from run 81 (see Supplementary Supplementary Table S1 online online) is shown. Note that symbolic communication did not emerge in this simulation run, i.e., agent communications did not evolve past indexical.

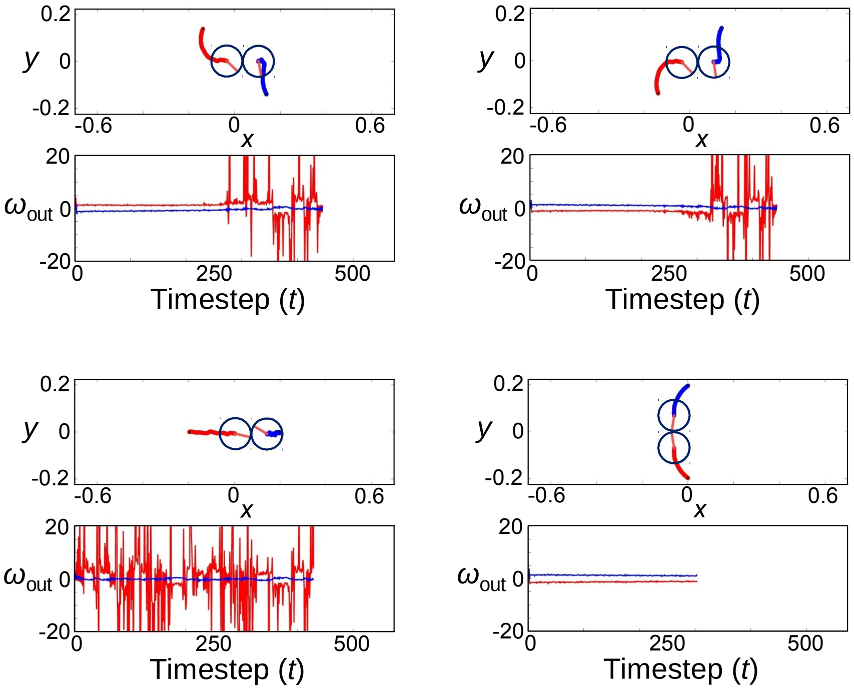

Figure S4. Additional sample behaviours from the agent analyzed in the main paper. Two clones of a top agent from run 7 (the run analyzed in the main body of the paper, see Supplementary Supplementary Table S1 online online) are tested from four different initial configurations and started simultaneously. The communication outputs and agent movements for these four test runs are shown.

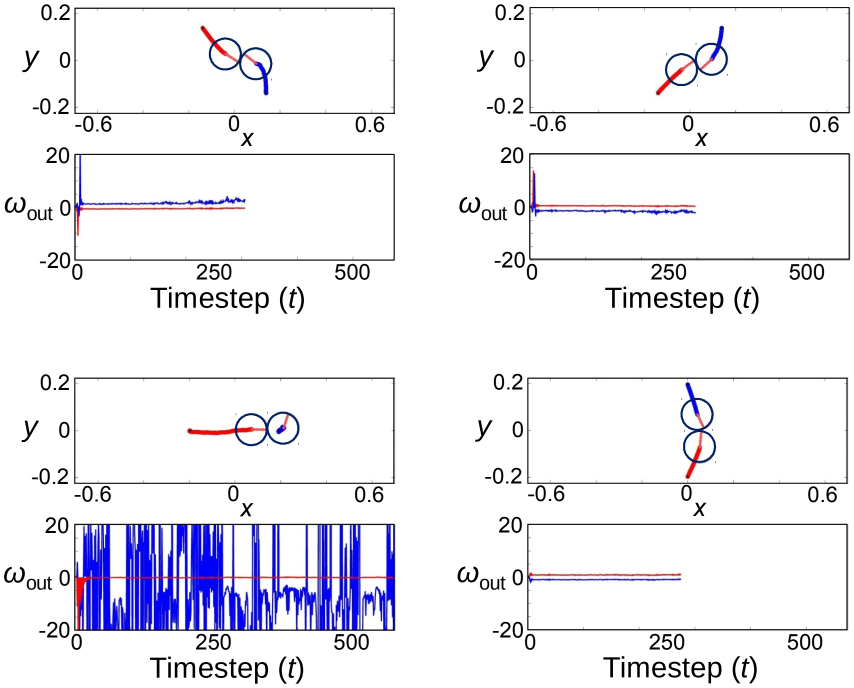

Figure S5. Sample behaviours from another evolutionary run. Two clones of a top agent from run 1 (see Supplementary Supplementary Table S1 online online) are tested from four different initial configurations and started simultaneously. The communication outputs and agent movements for these four test runs are shown.

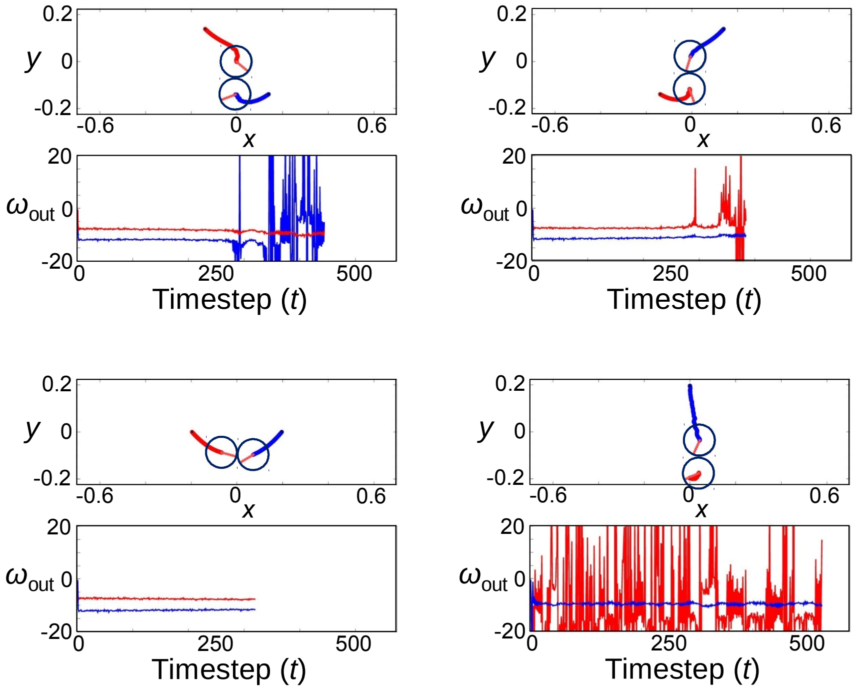

Figure S6. **Sample behaviours from another evolutionary run.** Two clones of a top agent from run 6 (see Supplementary Supplementary Table S1 online online) are tested from four different initial configurations and started simultaneously. The communication outputs and agent movements for these four test runs are shown.

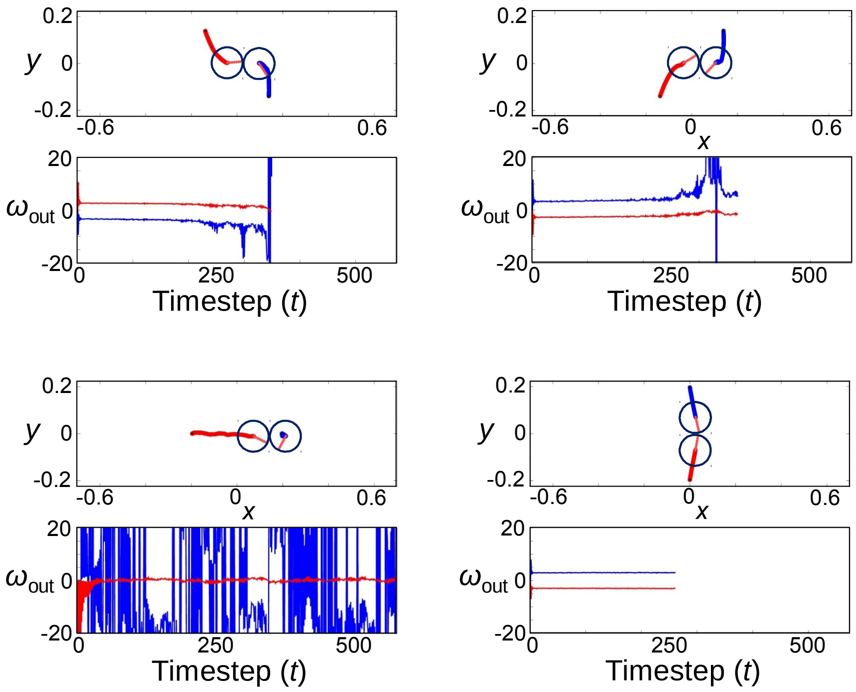

Figure S7. **Sample behaviours from another evolutionary run.** Two clones of a top agent from run 94 (see Supplementary Supplementary Table S1 online online) are tested from four different initial configurations and started simultaneously. The communication outputs and agent movements for these four test runs are shown.

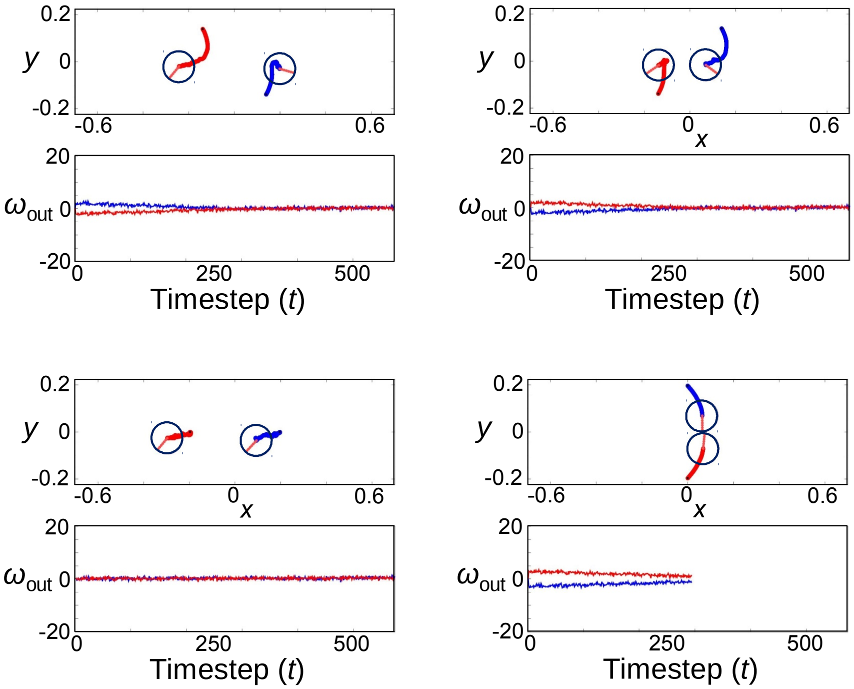

Figure S8. **Sample behaviours from another evolutionary run.** Two clones of a top agent from run 81 (see Supplementary Supplementary Table S1 online online) are tested from four different initial configurations and started simultaneously. The communication outputs and agent movements for these four test runs are shown. Note that symbolic communication did not emerge in this simulation run, i.e., agent communications did not evolve past indexical.

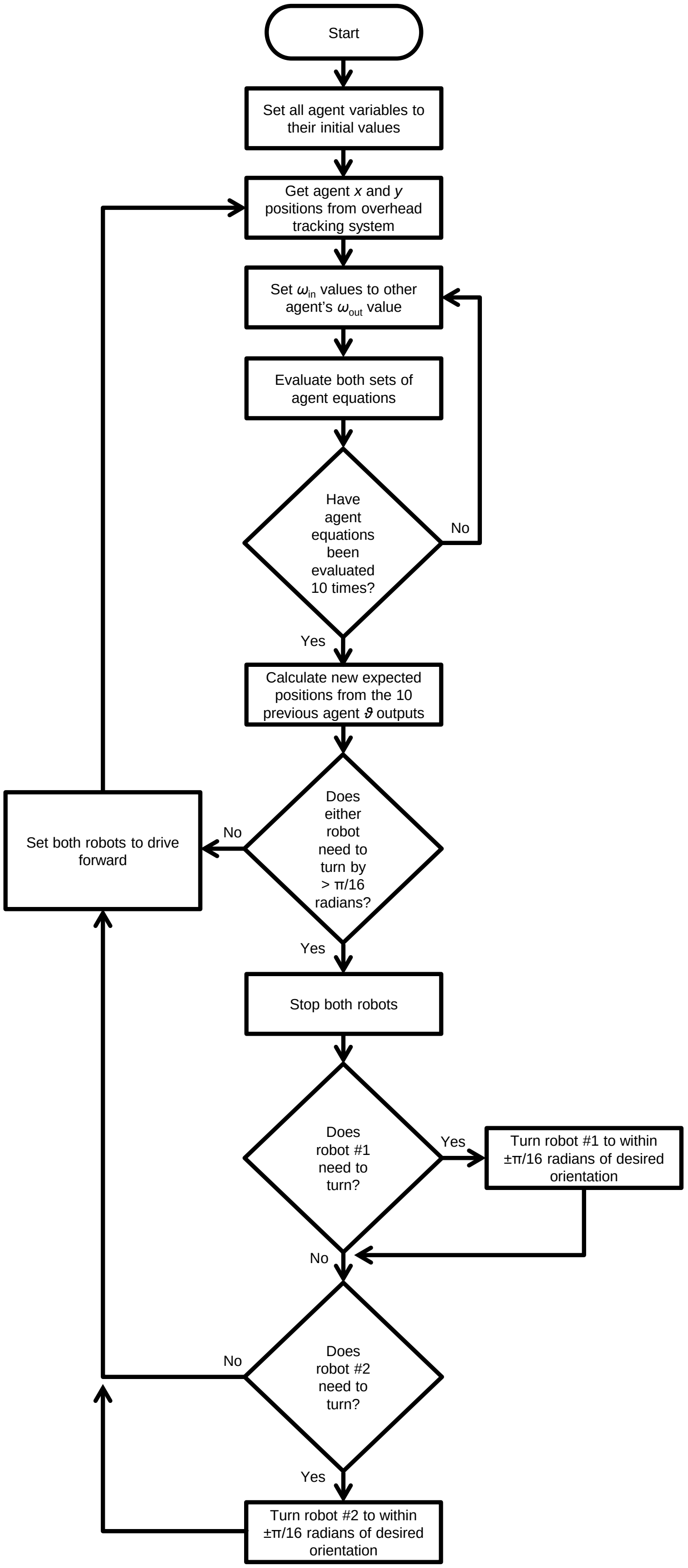

Figure S9. A flowchart of the protocol for the robotic experiments.

Supplementary Equations

The simplified and reduced evolved equation of the top agent from era 48 during run 7 is

$$\theta^{t+\Delta t} = \theta^t + v_1^t \tag{S1}$$

$$\omega_{\text{out}}^{t+\Delta t} = 3.74y^t \tag{S2}$$

$$v_1^{t+\Delta t} = \frac{3.82\,(y^t - 4.81)}{(\theta^t - 2.46)\left(0.67y^t + \theta^t + v_1^t - 3.21\right)} \tag{S3}$$

Communication has yet to emerge. Note that ω_{in} does not appear anywhere in the agent's governing equations. This is the run analyzed in the main text.

The simplified and reduced evolved equation of the top agent from era 313 during run 7 is

$$\theta^{t+\Delta t} = 2.45\left(\omega_{\text{in}}^t - 4.36y^t\right) \tag{S4}$$

$$\omega_{\text{out}}^{t+\Delta t} = 4.36y^t \tag{S5}$$

Indexical communication has emerged. This is the run analyzed in the main text.

The simplified and reduced evolved equation of the top agent from run 7 is

$$\theta^{t+\Delta t} = -5.50y^t + \omega_{\text{in}}^t \tag{S6}$$

$$\omega_{\text{out}}^{t+\Delta t} = 5.50y^t - \frac{1.39}{(\omega_{\text{in}}^t - 5.50y^t)(1.53 + x^t)} \tag{S7}$$

Symbolic communication has emerged, see Supplementary Table S1 online. Example behaviors can be found in Figs. 2, 3 and Supplementary Fig. S4 online. This is the symbolic agent analyzed in the main text.

The simplified and reduced evolved equation of the top agent from run 1 is

$$\theta^{t+\Delta t} = \omega_{\text{out}}^t + 0.91y^t - \frac{v_1^t}{\omega_{\text{out}}^t - \omega_{\text{in}}^t - 0.03} \tag{S8}$$

$$\omega_{\text{out}}^{t+\Delta t} = -0.06 - y^t - \frac{v_1^t}{-0.06 - y^t - \left(2.29/v_2^t v_5^t\right) - \omega_{\text{in}}^t} \tag{S9}$$

$$v_1^{t+\Delta t} = \frac{1.18}{-0.14 - (2.33/v_2^t)} \tag{S10}$$

$$v_2^{t+\Delta t} = v_3^t \tag{S11}$$

$$v_3^{t+\Delta t} = 1.36v_4^t + 1.46 \tag{S12}$$

$$v_4^{t+\Delta t} = 1.12x^t + 0.12 \tag{S13}$$

$$v_5^{t+\Delta t} = (v_3^t + 7.81)(-3.42v_1^t + v_6^t + v_7^t) \tag{S14}$$

$$v_6^{t+\Delta t} = 0.40y^t - 8.57 - \frac{2.29}{v_2^t v_5^t} \tag{S15}$$

$$v_7^{t+\Delta t} = 10.22 + v_5^t \tag{S16}$$

Symbolic communication emerged, see Supplementary Table S1 online. Example behaviors can be found in Supplementary Fig. S5 online.

The simplified and reduced evolved equation of the top agent from run 5 is

$$\theta^{t+\Delta t} = \frac{v_1^t}{\omega_{\text{in}}^t + y^t} \tag{S17}$$

$$\omega_{\text{out}}^{t+\Delta t} = -y^t - \frac{2.14v_1^t}{(x^t - 2.79)(v_2^t - 1.38)(y^t + \omega_{\text{in}}^t)} \tag{S18}$$

$$v_1^{t+\Delta t} = -0.50x^t - 0.80 \tag{S19}$$

$$v_2^{t+\Delta t} = -x^t + 3.92 \tag{S20}$$

Symbolic communication emerged, see Supplementary Table S1 online.

The simplified and reduced evolved equation of the top agent from run 6 is

$$\theta^{t+\Delta t} = -1.59 + \frac{-0.76 + 1.58\,(y^t - 1.40)}{8.57\,(1.15 + x^t) + \omega_{\text{in}}^t} + \frac{0.22v_4^t((x^t - v_5^t - 1.71)/(v_2^t v_8^t))}{8.57\,(1.15 + x^t) + \omega_{\text{in}}^t} \tag{S21}$$

$$\omega_{\text{out}}^{t+\Delta t} = -8.42\left(1.15 + x^t\right) + \frac{v_1^t}{8.42\,(1.15 + x^t) + \omega_{\text{in}}^t} \tag{S22}$$

$$v_1^{t+\Delta t} = 2.51\left(y^t - 1.22\right) \tag{S23}$$

$$v_2^{t+\Delta t} = 57.13x^t + 0.78v_2^t + 0.78v_6^t + 58.07 - \frac{0.81v_1^t}{x^t + 0.12v_7^t + 1.15} \tag{S24}$$

$$v_3^{t+\Delta t} = \frac{v_1^t}{\omega_{\text{out}}^t} \tag{S25}$$

$$v_4^{t+\Delta t} = v_3^t - \frac{v_1^t}{\omega_{\text{out}}^t} \tag{S26}$$

$$v_5^{t+\Delta t} = v_3^t\left(\frac{v_1^t}{\omega_{\text{out}}^t} - \frac{1.97(-x^t + v_5^t + 1.71)}{v_2^t}\right) \tag{S27}$$

$$v_6^{t+\Delta t} = v_2^t \tag{S28}$$

$$v_7^{t+\Delta t} = v_4^t + \omega_{\text{in}}^t - v_6^t \tag{S29}$$

$$v_8^{t+\Delta t} = \frac{0.75(v_8^t - 0.59)}{\omega_{\text{out}}^t} \tag{S30}$$

Symbolic communication emerged, see Supplementary Table S1 online. Example behaviors can be found in Supplementary Fig. S6 online.

The simplified and reduced evolved equation of the top agent from run 24 is

$$\theta^{t+\Delta t} = 3.06x^t - \omega_{\text{in}}^t \tag{S31}$$

$$\omega_{\text{out}}^{t+\Delta t} = 3.06x^t + \frac{8.88}{\left(3.06x^t - \omega_{\text{in}}^t\right)(3.00y^t + 6.36)} \tag{S32}$$

Symbolic communication emerged, see Supplementary Table S1 online. Note that this simulation run was done with θ^t measured relative to "north" ($+y$ direction), instead of relative to "east" ($+x$ direction). All other simulation details remain unchanged.

The simplified and reduced evolved equation of the top agent from run 33 is

$$\theta^{t+\Delta t} = \frac{v_1^t}{9.91x^t - \omega_{\text{in}}^t} \tag{S33}$$

$$\omega_{\text{out}}^{t+\Delta t} = 9.91x^t - \frac{2.00\theta^t}{v_3^t - 2.06} \tag{S34}$$

$$v_1^{t+\Delta t} = v_2^t \tag{S35}$$

$$v_2^{t+\Delta t} = y^t + v_3^t + 3.45 \tag{S36}$$

$$v_3^{t+\Delta t} = y^t \tag{S37}$$

Symbolic communication emerged, see Supplementary Table S1 online. Note that this simulation run was done with θ^t measured relative to "north" ($+y$ direction), instead of relative to "east" ($+x$ direction). All other simulation details remain unchanged.

The simplified and reduced evolved equation of the top agent from run 50 is

$$\theta^{t+\Delta t} = -\frac{2.59}{v_3^t\left(3.63 - v_6^t\right)} \tag{S38}$$

$$\omega_{\text{out}}^{t+\Delta t} = y^t + 2.75 - \frac{0.08v_1^t}{(x^t + 1.43)\left(\omega_{\text{in}}^t - y^t - 2.74\right)} \tag{S39}$$

$$v_1^{t+\Delta t} = v_2^t \tag{S40}$$

$$v_2^{t+\Delta t} = 2.07 - x^t \tag{S41}$$

$$v_3^{t+\Delta t} = \frac{0.91}{\left(v_4^t - 12.69\right)\left(\omega_{\text{in}}^t - y^t - 2.74\right)} \tag{S42}$$

$$v_4^{t+\Delta t} = v_5^t \tag{S43}$$

$$v_5^{t+\Delta t} = v_1^t + 6.77 - \frac{0.74}{v_2^t} \tag{S44}$$

Symbolic communication emerged, see Supplementary Table S1 online.

The simplified and reduced evolved equation of the top agent from run 70 is

$$\theta^{t+\Delta t} = \frac{3.49x^t + v_1^t + 5.48}{-12.13y^t - \omega_{\text{in}}^t - v_3^t} \tag{S45}$$

$$\omega_{\text{out}}^{t+\Delta t} = \theta^t - 11.96y^t + \frac{4.06x^t + 5.04}{-11.91y^t - \omega_{\text{in}}^t - v_1^t v_2^t} \tag{S46}$$

$$v_1^{t+\Delta t} = v_4^t \tag{S47}$$

$$v_2^{t+\Delta t} = \frac{0.12\left(y^t\right)^2}{\left(2.93/v_2^t\right) - 2.09} \tag{S48}$$

$$v_3^{t+\Delta t} = -1.37v_2^t \tag{S49}$$

$$v_4^{t+\Delta t} = -v_2^t - 0.11 \tag{S50}$$

Symbolic communication emerged, see Supplementary Table S1 online.

The simplified and reduced evolved equation of the top agent from run 94 is

$$\theta^{t+\Delta t} = -\frac{3.43}{y^t - \frac{x^t - 3.39}{y^t + \frac{4.07}{y^t + \frac{4.63}{y^t + \frac{4.38}{y^t - \omega_{\text{in}}^t}}}}} \tag{S51}$$

$$\omega_{\text{out}}^{t+\Delta t} = y^t + \frac{3.39}{y^t + \frac{3.96}{y^t + \frac{6.51}{y^t - \frac{x^t - 3.96}{y^t + \frac{4.73}{y^t - \frac{x^t - 3.48}{y^t + \frac{5.31}{y^t + \frac{4.11}{y^t + \frac{3.65}{y^t - \omega_{\text{in}}^t}}}}}}}}} \tag{S52}$$

Symbolic communication emerged, see Supplementary Table S1 online. Example behaviors can be found in Supplementary Fig. S7 online.

The simplified and reduced evolved equation of the top agent from run 81 is

$$\theta^{t+\Delta t} = \frac{5.34}{\omega_{\text{out}}^t - \omega_{\text{in}}^t + \left(5.34/\left(\theta^t + \omega_{\text{out}}^t - \omega_{\text{in}}^t - 0.20\right)\right)} \tag{S53}$$

$$\omega_{\text{out}}^{t+\Delta t} = -14.36 y^t \tag{S54}$$

Symbolic communication did not emerge, this is an example of an agent that employs indexical communication, see Supplementary Table S1 online. Example behaviors can be found in Supplementary Fig. S8 online.

Supplementary Video Captions

Video S1: Embodiment experiments on e-puck robotic hardware. This video shows hardware experiments from three different initial configurations using two clones of a top agent from a *NoiseWorld* run in which symbolic communication emerged (see Methods and Fig. 4 for details). The audio was generated from the agents' numerical communications (i.e., each agent's ω_{out}, see Methods). "Big Red" refers to the robot with the biggest red square (used for overhead position detection), while "Big Blue" refers to the other robot (biggest blue square).

Video S2: Emergence of symbolic communication from within a population of initially noncommunicating robots. This video summarizes our experimental setup and simulation and hardware results.

Run Number	Max. Island Reproduction Rate (per era)	Total Number of Eras	Symbolic Comm. Emerged?	Run Number	Max. Island Reproduction Rate (per era)	Total Number of Eras	Symbolic Comm. Emerged?
1	23072	1014	Yes	56	17590	1381	No
2	20798	1269	No	57	21862	914	No
3	21575	1325	No	58	17333	1735	No
4	17580	1489	No	59	16732	1163	No
5	25295	1408	Yes	60	21823	1609	No
6	25281	1038	Yes	61	20310	1428	No
7*	23394	1430	Yes	62	21089	1214	No
8	21302	1476	No	63	20048	1662	No
9	20447	1393	No	64	20581	1432	No
10	20969	1519	No	65	20750	1462	No
11	21306	1045	No	66	17679	1698	No
12	21519	1090	No	67	21215	1458	No
13	19158	1092	No	68	21249	1627	No
14	17729	1460	No	69	21868	1276	No
15	20891	1580	No	70	23400	1247	Yes
16	20290	1214	No	71	20071	1451	No
17	20982	1378	No	72	20502	1061	No
18	17450	2081	No	73	21665	1113	No
19	21283	1273	No	74	18023	1358	No
20	18654	1237	No	75	20477	1677	No
21†	20900	1477	No	76	20574	1498	No
22†	20324	1110	No	77	17637	1548	No
23†	20928	1346	No	78	21501	997	No
24†	22994	1069	Yes	79	18328	1679	No
25†	22000	1366	No	80	18043	1454	No
26†	19895	1051	No	81	21111	1613	No
27†	19311	849	No	82	21041	980	No
28†	17397	1854	No	83	19896	1352	No
29†	20313	1116	No	84	21019	1542	No
30†	21491	1562	No	85	21110	1680	No
31†	21725	1342	No	86	20517	1854	No
32†	21158	1364	No	87	20341	1402	No
33†	22606	1455	Yes	88	20565	1210	No
34†	17228	1872	No	89	21039	1043	No
35†	21051	713	No	90	20503	1337	No
36†	16487	956	No	91	20163	1063	No
37†	21212	1358	No	92	21172	1206	No
38†	20051	1850	No	93	20032	977	No
39†	20834	1305	No	94	23982	1152	Yes
40†	17901	1576	No	95	19814	1178	No
41	18649	1469	No	96	20775	1538	No
42	21367	1443	No	97	18095	1792	No
43	21097	1686	No	98	21240	1292	No
44	20902	597	No	99	21436	995	No
45	20691	793	No	100	16863	1748	No
46	21707	1412	No	101§	7417	1074	N/A
47	20874	1863	No	102§	7027	1099	N/A
48	20513	1263	No	103§	7279	1192	N/A
49	18107	1822	No	104§	7118	1458	N/A
50	24987	1293	Yes	105§	8076	1009	N/A
51	19933	1012	No	106§	7765	1173	N/A
52	17385	1677	No	107§	7547	1064	N/A
53	17456	1901	No	108§	7245	1086	N/A
54‡	8383	1007	No	109§	7671	1699	N/A
55	18278	964	No	110§	7627	1585	N/A

**Table S1. Data from 110 simulation runs. (***) This simulation run is the one that was analyzed in the main body of this paper. (†) These simulation runs were done with ϑ^t measured relative to “north” (+*y* direction), instead of relative to “east” (+*x* direction). All other simulation details remain unchanged. (‡) Communication did not emerge during this simulation run. (§) Communication was disabled in these runs, i.e., $\omega_{in} = 0$ for all *t*.

Table S2. Data from addition simulation runs with migration disabled.

Migration allowed?	Number of runs	Number to reach symbolic	Max. isl. repro. rate (per era)			Number of eras		
			μ	σ	Rank-sum	μ	σ	Rank-sum
Yes	100	9	20261.74	2192.90		1357.00	290.84	
					P < 0.00001			*P* = 0.00960
No[*]	50	1	17784.60	1174.14		1267.50	55.20	
					P = 0.03010			*P* < 0.00001
No[†]	50	0	17303.42	1076.78		676.32	29.70	

Here we compare an additional 50 experiments with migration disabled to the first 100 reported experiments. Simulations run without migration were significantly slower; therefore two 48 hour runs were needed to reach a similar number of eras as the original experiments.

* These are the results after 96 wall clock hours of simulation.

† There are the results after the first 48 wall clock hours of simulation.
